# Supplementary material for: Resolving the full spectrum of human genome variation using Linked-Reads
Source: Genome Res. 2019 Apr;29(4):635–45. doi: 10.1101/gr.234443.118 (PMC6442396; doi:10.1101/gr.234443.118)
Supplement: Supplemental Material [file supp_gr.234443.118_Supplemental_longranger-2.2.zip › longranger-2.2.tar.bz2!/lr2.2-public/lib/stan/cmdstan/stan/doc/api/html/structstan_1_1gm_1_1is__nil__op.html]

Stan: stan::gm::is\_nil\_op Struct Reference


|  |  |
| --- | --- |
|  | Stan  2.5.0  probability, sampling & optimization |


- Main Page
- Related Pages
- Namespaces
- Classes
- Files

- Class List
- Class Index
- Class Hierarchy
- Class Members

All Classes Namespaces Files Functions Variables Typedefs Enumerations Enumerator Friends Macros Pages

- stan
- gm
- is\_nil\_op

Public Member Functions |
List of all members

stan::gm::is\_nil\_op Struct Reference

`#include <ast.hpp>`

Inheritance diagram for stan::gm::is\_nil\_op:

|  |  |
| --- | --- |
| Public Member Functions | |
| bool | operator() (const nil &x) const |
|  | |
| bool | operator() (const int\_literal &x) const |
|  | |
| bool | operator() (const double\_literal &x) const |
|  | |
| bool | operator() (const array\_literal &x) const |
|  | |
| bool | operator() (const variable &x) const |
|  | |
| bool | operator() (const integrate\_ode &x) const |
|  | |
| bool | operator() (const fun &x) const |
|  | |
| bool | operator() (const index\_op &x) const |
|  | |
| bool | operator() (const binary\_op &x) const |
|  | |
| bool | operator() (const unary\_op &x) const |
|  | |

## Detailed Description

Definition at line 270 of file ast.hpp.

## Member Function Documentation

|  |  |  |  |  |  |
| --- | --- | --- | --- | --- | --- |
| bool stan::gm::is\_nil\_op::operator() | ( | const nil & | *x* | ) | const |

Definition at line 754 of file ast\_def.cpp.

|  |  |  |  |  |  |
| --- | --- | --- | --- | --- | --- |
| bool stan::gm::is\_nil\_op::operator() | ( | const int\_literal & | *x* | ) | const |

Definition at line 755 of file ast\_def.cpp.

|  |  |  |  |  |  |
| --- | --- | --- | --- | --- | --- |
| bool stan::gm::is\_nil\_op::operator() | ( | const double\_literal & | *x* | ) | const |

Definition at line 756 of file ast\_def.cpp.

|  |  |  |  |  |  |
| --- | --- | --- | --- | --- | --- |
| bool stan::gm::is\_nil\_op::operator() | ( | const array\_literal & | *x* | ) | const |

Definition at line 757 of file ast\_def.cpp.

|  |  |  |  |  |  |
| --- | --- | --- | --- | --- | --- |
| bool stan::gm::is\_nil\_op::operator() | ( | const variable & | *x* | ) | const |

Definition at line 758 of file ast\_def.cpp.

|  |  |  |  |  |  |
| --- | --- | --- | --- | --- | --- |
| bool stan::gm::is\_nil\_op::operator() | ( | const integrate\_ode & | *x* | ) | const |

Definition at line 759 of file ast\_def.cpp.

|  |  |  |  |  |  |
| --- | --- | --- | --- | --- | --- |
| bool stan::gm::is\_nil\_op::operator() | ( | const fun & | *x* | ) | const |

Definition at line 760 of file ast\_def.cpp.

|  |  |  |  |  |  |
| --- | --- | --- | --- | --- | --- |
| bool stan::gm::is\_nil\_op::operator() | ( | const index\_op & | *x* | ) | const |

Definition at line 761 of file ast\_def.cpp.

|  |  |  |  |  |  |
| --- | --- | --- | --- | --- | --- |
| bool stan::gm::is\_nil\_op::operator() | ( | const binary\_op & | *x* | ) | const |

Definition at line 762 of file ast\_def.cpp.

|  |  |  |  |  |  |
| --- | --- | --- | --- | --- | --- |
| bool stan::gm::is\_nil\_op::operator() | ( | const unary\_op & | *x* | ) | const |

Definition at line 763 of file ast\_def.cpp.

---

The documentation for this struct was generated from the following files:

- src/stan/gm/ast.hpp
- src/stan/gm/ast\_def.cpp


---

[ Stan Home Page ]

*© 2011–2014,
Stan Development Team.*
